# Annual dynamic dataset of global cropping intensity from 2001 to 2019

**DOI:** 10.1038/s41597-021-01065-9

**Published:** 2021-10-28

**Authors:** Xiaoxuan Liu, Juepeng Zheng, Le Yu, Pengyu Hao, Bin Chen, Qinchuan Xin, Haohuan Fu, Peng Gong

**Affiliations:** 1 Ministry of Education Key Laboratory for Earth System Modeling, Department of Earth System Science, Tsinghua University, Beijing, 100084 China; 2 Aerospace Information Research Institute, Chinese Academy of Sciences, Beijing, 100190 China; 3 Ministry of Education Ecological Field Station for East Asia Migratory Birds, Tsinghua University, Beijing, 100084 China; 4 Center for Spatial Information Science and Systems, George Mason University, Fairfax, Virginia USA; 5 Department of Land, Air and Water Resources, University of California, Davis, CA 95616 USA; 6 School of Geography and Planning, Sun Yat-sen University, Guangzhou, 510275 China; 7 National Supercomputing Center in Wuxi, Wuxi, 214000 China; 8 Department of Geography and Department of Earth Sciences, University of Hong Kong, Hong Kong, China

**Keywords:** Agroecology, Phenology

## Abstract

The cropping intensity has received growing concern in the agriculture field in applications such as harvest area research. Notwithstanding the significant amount of existing literature on local cropping intensities, research considering global datasets appears to be limited in spatial resolution and precision. In this paper, we present an annual dynamic global cropping intensity dataset covering the period from 2001 to 2019 at a 250-m resolution with an average overall accuracy of 89%, exceeding the accuracy of the current annual dynamic global cropping intensity data at a 500-m resolution. We used the enhanced vegetation index (EVI) of MOD13Q1 as the database via a sixth-order polynomial function to calculate the cropping intensity. The global cropping intensity dataset was packaged in the GeoTIFF file type, with the quality control band in the same format. The dataset fills the vacancy of medium-resolution, global-scale annual cropping intensity data and provides an improved map for further global yield estimations and food security analyses.

## Abstract (summary)

**Table:** 

Measurement(s)	cropping intensity
Technology Type(s)	sixth-order polynomial function
Factor Type(s)	temporal interval • geographic location
Sample Characteristic - Environment	cultivated environment
Sample Characteristic - Location	global

Machine-accessible metadata file describing the reported data: 10.6084/m9.figshare.15128076

## Background & Summary

Croplands are rapidly changing under the high-intensity management of human activities^1^. In contrast to other natural vegetation for which the growth process is completed across multiple years, like forests or grasslands, croplands complete their growth process within a year, and this process is more comprehensive because of phenological changes under different cropping systems in different regions^2,3^. Moreover, the distribution characteristics and state of croplands are strongly affected by human beings, such as through the use of multiple cropping systems and field management^4^. The multiple cropping intensity is a significant indicator used to measure multi-cropping systems the utilization of agricultural land resources^5^. To ensure national food security, the importance of systematic research on the utilization and change trends of land intensification has become increasingly prominent and is of great significance for guiding agricultural production and obtaining a deep understanding of dynamic cropland changes^6,7^. Numerous researchers have developed global or regional cropping intensity maps using remote sensing datasets or statistical datasets. Some of these maps focus on the national scale while ignoring spatial information;^8,9^ some emphasize the corresponding algorithm while only experimenting in a few selected regions;^10,11^ and a few researchers have given close attention to studying the global cropping intensity but only in a single year^4,6,12^, and these datasets might be difficult to use in annual dynamic monitoring applications^13^. The MODIS Land Cover Dynamics Product (MCD12Q2) provides a global NumCycles dataset at a 500-m spatial resolution and an annual time step; while this product comprises all land cover types, it does not consider the cropping intensity^14^. Notwithstanding previous studies, due to the need for a finer resolution and a more precise dataset, a study in which the cropping intensity can be updated quickly in a simple manner would be useful for future cropland analyses and food security estimations.

In this paper, we used the enhanced vegetation index (EVI) data of MOD13Q1 to build a database to fit the whole phenological process within the cropland extent. To produce the dataset quickly, we introduced a sixth-order polynomial function to calculate the cropping intensity because of its rapidity and versatility. The dataset described the cropping intensity distribution and its qualitative value from 2001 to 2019 at a 250-m resolution with an average overall accuracy of 89%; these data were packaged in the GeoTIFF file type, and the quality control band was in the same format. This global cropping intensity dataset provides an improved map for further global yield estimations and food security analyses^5,15^.

## Methods

### Data collection

Based on the cropland extent, we first introduced a cropland distribution template, the Self-adapting Statistics Allocation Model of Global Cropland (SASAM-GC)^16^, as shown in Fig. S1. The global cropland extent map used herein was a global cropland synergy map with a 500-m spatial resolution representing approximately the year 2010, developed by the Smart Agriculture Innovation Team of the Key Laboratory of Agricultural Remote Sensing (AGRIRS) of the Chinese Academy of Agricultural Sciences in cooperation with the International Food Policy Research Institute (IFPRI) and the International Institute of Applied Systems Analysis (IIASA). The overall accuracy of the SASAM-GC products is 90.8%, which is higher than those of existing global farmland products such as the Climate Change Initiative Land Cover (CCI-LC), GlobeLand30, and Medium Resolution Imaging Spectrometer (MODIS) products.

Vegetation indices are often used to depict crop growth, such as the ratio vegetation index (RVI)^17^, normalized difference vegetation index (NDVI), and enhanced vegetation index (EVI)^18^. Among them, the EVI is the most sensitive to high-biomass regions and less susceptible to atmospheric and soil interference^19,20^. MODIS vegetation index datasets are generated every 8 days or 16 days at spatial resolutions of 250 m, 500 m, and 1000 m. The 250-m spatial resolution is considered the best resolution for detecting crops^21,22^. Here, we used EVI time series with a spatial resolution of 250 m reported every 16 days in the MODIS product MOD13Q1 as the primary data to calculate the global cropping intensity; these data can be accessed at https://lpdaac.usgs.gov/products/mod13q1v006/.

1 $${\rm{EVI}}=2.5\times \frac{{{\rm{\rho }}}_{{\rm{NIR}}}-{{\rm{\rho }}}_{{\rm{Red}}}}{{{\rm{\rho }}}_{{\rm{NIR}}}+6\times {{\rm{\rho }}}_{{\rm{Red}}}-7.5\times {{\rm{\rho }}}_{{\rm{Blue}}}+1}$$

In formula 1, ρ_NIR_, ρ_Red_, and ρ_Blue_ represent the reflectivity of the near-infrared band, the red band, and the blue band, respectively.

The Food and Agricultural Organization of the United Nations statistical data (FAOSTAT) provides long-time-series cropland-related statistical data at the country level and can be accessed at http://www.fao.org/faostat/en/#data. FAOSTAT cropland data have been widely used in a variety of studies evaluating food security and hindcasting historical land-use changes^23,24^. Here, we defined the cropland area as the sum of areas characterizing arable land (land under temporary crops, temporary meadows used for mowing or pasture, market and kitchen gardens, and land that is temporarily fallow; abandoned land resulting from shifting cultivation was excluded) and permanent croplands (land cultivated with long-term crops that do not have to be replanted for several years). Additionally, the harvested area is used; this value refers to the area from which a crop is gathered. If the crop under consideration is harvested more than once during a year due to successive cropping (i.e., the same crop is sown or planted more than once in the same field during the same year), the area is counted as many times as the crop is harvested. Therefore, the sown area is recorded only for the crop reported. The statistical cropping intensity is defined as the harvested area divided by the cropland area and is used to validate the consistency between the cropping intensity obtained herein and that reported in the FAOSTAT product at the country level.

### Brief algorithm

In this study, a sixth-order polynomial function was used to reconstruct EVI time series for brief and rapid calculations at the global scale^25^. As the global cropping intensity ranges from 0 to 3 and the sixth-order polynomial function can have 3 maxima at most, we chose the sixth-order polynomial function (formula 2) in this study:

2 $${\rm{EVI}}({\rm{t}})={{\rm{a}}}_{0}+{{\rm{a}}}_{1}{\rm{t}}+{{\rm{a}}}_{2}{{\rm{t}}}^{2}+\cdots +{{\rm{a}}}_{6}{{\rm{t}}}^{6}$$

where t is the time-series length referring to the beginning of the time series, EVI(t) is the fitted EVI time series, and a_0_, a_1_, … a_n_ are the fitted parameters of the sixth-order polynomial function. Then, the first derivative EVI′(t) and the second derivative EVI″(t) were calculated to find the real numerical solution of the equation’s maxima when EVI′(t) = 0and EVI″(t) < 0.

**Fig. 1:**
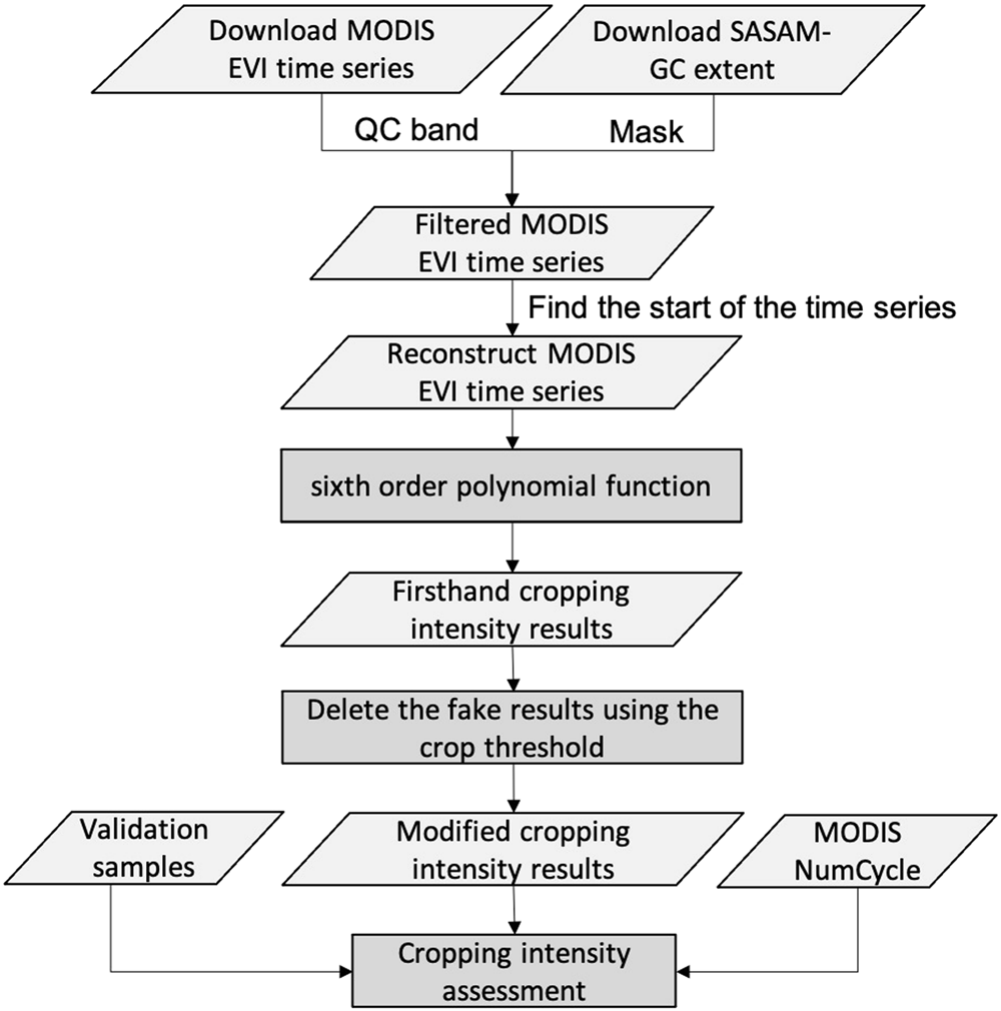
Schematic overview of the cropping intensity production workflow.

**Fig. 2:**
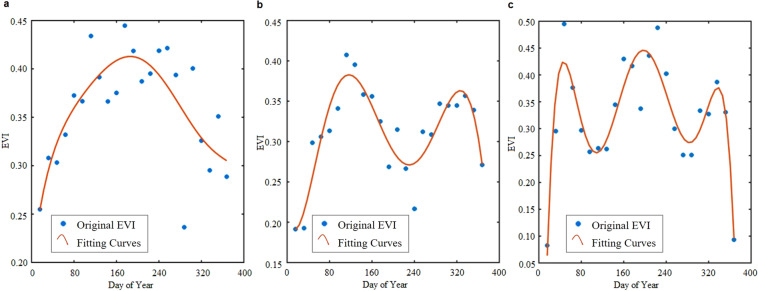
Examples of the results of cropping intensity calculations via a sixth-order polynomial function: (**a**) single cropping intensity, (**b**) double cropping intensity, and (**c**) triple cropping intensity.

The procedures followed herein to obtain the global cropping intensity calculation are outlined in Fig. 1:

We first extracted global MODIS EVI time series from 2000 to 2020 according to the SASAM-GC product with areas with cropland probabilities greater than 10% considered as the cropland extent. Moreover, within the global cropland extent, we used the quality control band (QC) to filter a better MODIS EVI time series and interpolate incorrect time values using time series linear interpolation (see Fig. S2 for an example). Using the EVI period from 2000 to 2020, we expanded the EVI time series of each year from 2001 to 2019 by the three months at the end of the previous year and the three months at the beginning of the next year to obtain a total of 18 months in each annual EVI time series. The first minimum value of each 18-month EVI time series was judged as the starting point of the phenological curve. Inconsistent locations were changed to denote the start of planting as the starting point, and a new, convex 12-month EVI time series was reconstructed for each year. Then, this convex EVI time series could be used with sixth-order polynomial fitting to meet the single-peak to three-peaks conditions, as shown in Fig. 2 for single-peak, double-peak, and triple-peak examples, representing multiple cropping intensities once, twice, and three times, respectively. According to the existing literature, filtering using EVI values with peaks value greater than 0.35, differences between the EVI peaks and valleys greater than 0.01, and durations between the peaks and valleys longer than three months could remove some false peaks caused by other vegetation^14,26^. Finally, we deleted the number of fake peaks and overfitted peaks according to the experiential threshold and identified the multiple cropping intensity.

According to the above steps, we calculated global time series of farmland pixels annually from 2001 to 2019 to obtain global multiple cropping index distribution maps (see Fig. S3). Examples of increasing and decreasing cropping intensities are also listed in Fig. S4.

### Quality control

To make it easy for users to apply our cropping intensity dataset, we introduced a QC band during the production period. The QC band contains four numbers, with values ranging from 0–3 corresponding to “best”, “good”, “fair” and “poor”. If a pixel is judged as “best”, it should satisfy all of the following conditions; “good” pixels meet two of these conditions, “fair” pixels meet one condition, and “poor” pixels meet none of them.

- Half of the annual EVI time series should be acceptable according to the MODIS EVI_QC band;
- The interpolation counts should be less than half of the whole EVI time series, for a total of 23 counts;
- We introduced the delta value here, an estimate of the standard error in predicting value t by EVI(t). The EVI(t) value ± delta should meet the cropping intensity identification conditions.

## Data Records

Global cropping intensity maps and QC band maps at a 250-m resolution were provided for the entire world from 2001 to 2019. The datasets and their validation samples are available at the figshare repository in GeoTIFF format and provided in the GCS_WGS_1984 spatial reference system^27^. The global cropping intensity maps contain values of 0, 1, 2, and 3, representing none, single, double, and triple cropping, respectively. The QC band maps also contain values of 0, 1, 2 and 3, representing best, good, fair, and poor pixels, respectively. The dataset extends from 70° N to 60° S latitude and from 180° W to 180° E longitude, excluding Greenland and Antarctica. The maps can be visualized and analyzed in ArcGIS, QGIS, or similar software.

## Technical Validation

### Verifying the precision using the validation samples

**Fig. 3:**
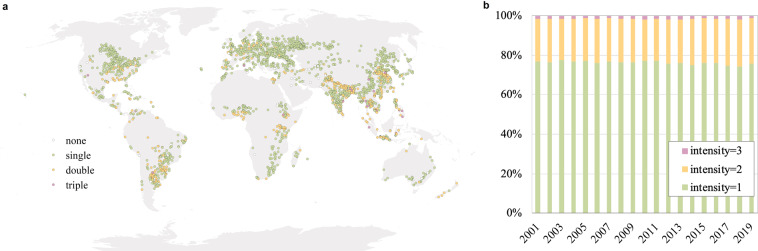
(**a**) Validation sample distribution in 2001, for example; (**b**) proportions of cropping intensity values among the validation samples, including single, double and triple cropping intensities, from 2001 to 2019.

To further the application credibility of the dataset, we introduced validation samples, comprising 2492 annual samples from 2001 to 2019, to validate our GCI (Global Cropping Intensity) datasets. These samples are all well-qualified cropland types based on the Finer Resolution Observation and Monitoring of Global Land Cover (FROM-GLC)^28–30^ product obtained under an effective manual interpretation of their EVI time series curve. Figure 3 is an example of samples obtained in 2001 and all cropping intensity value statistics from 2001 to 2019.

**Fig. 4:**
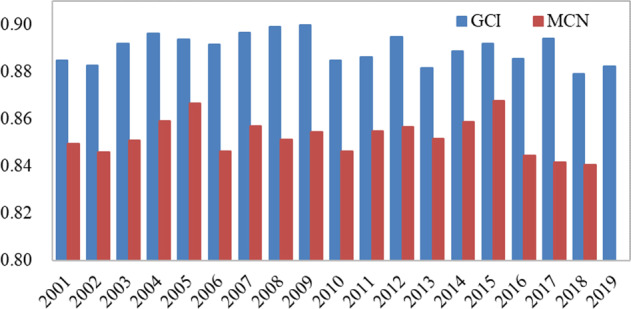
The overall accuracies of the GCI and MCI datasets from 2001 to 2019.

An accuracy assessment of the refined cropland extent in each study region was conducted by calculating the overall accuracy (OA) from the confusion matrix. As shown by the blue bar in Fig. 4, we found that the cropping intensity dataset from 2001 to 2019 had a good overall accuracy performance, with an average OA of 88.97% and a maximum precision of 89.97%. In general, cropping intensities with fewer cycles show better accuracy than multiple-cropping intensities.

### Comparison of the results with MODIS CycleNum

For further analysis of our cropping intensity dataset, we introduced the MCD12Q2 CycleNum Dataset for comparison, although this dataset has a coarser spatial resolution; thus, we upscaled our results to a 500-m spatial resolution to match that of the MCD12Q2 product. Figure 4 exhibits the overall accuracies obtained for our cropping intensity dataset (GCI hereinafter) and MCD12Q2 CycleNum (MCN hereinafter) according to our global validation samples. In general, we found that the accuracies of GCI and MCN show good performances, with both OAs over 80%. Separately, the OAs of GCI ranged from 87.92% (2018) to 89.97% (2009), while the OAs of MCN ranged from 84.04% (2018) to 86.76% (2015); these values show the better accuracy of our new global cropping intensity dataset. Compared to the MCN dataset, our GCI dataset has a significant improvement of nearly 5% OA calculated with z-score statistics, proving our dual improvement in both the spatial resolution and overall accuracy of the dataset.

**Fig. 5:**
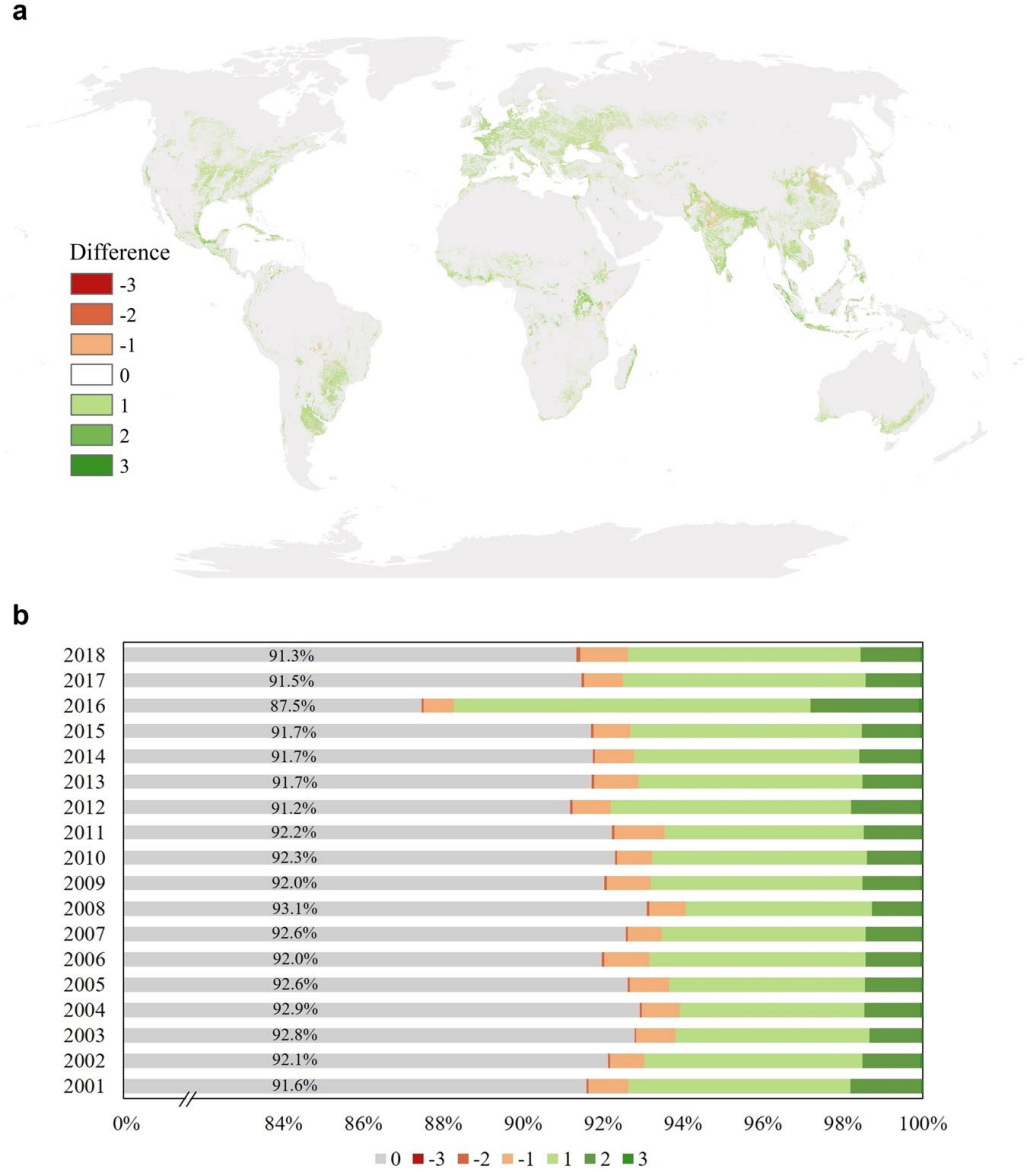
(**a**) The spatial distribution between the GCI and MCN datasets; (**b**) statistics of the cropping intensity differences between the GCI and MCN datasets.

Additionally, the spatial distribution difference between the MCN dataset and our global cropping intensity dataset was used to compare the spatial dimensions of these datasets. In Fig. 5(a), a positive value indicates that the GCI result output a higher number than the MCN product, while a negative value indicates an underestimation by our dataset. Although there is a large-scale distribution of positive numbers and the cropping intensity difference ranges from −3 to 3 in the global map (see Fig. 5(a)), we found that the GCI dataset is generally consistent with the MCN. Figure 5(b) displays the cropping intensity difference between the GCI and MCN datasets, indicating no difference for most areas (over 90% for nearly all years of study) within the cropland extent. Moreover, the average difference values in all years are less than 1, illustrating an evident agreement between the GCI and MCN datasets and demonstrating a more conservative estimation by the MCN dataset than by the GCI dataset.

**Fig. 6:**
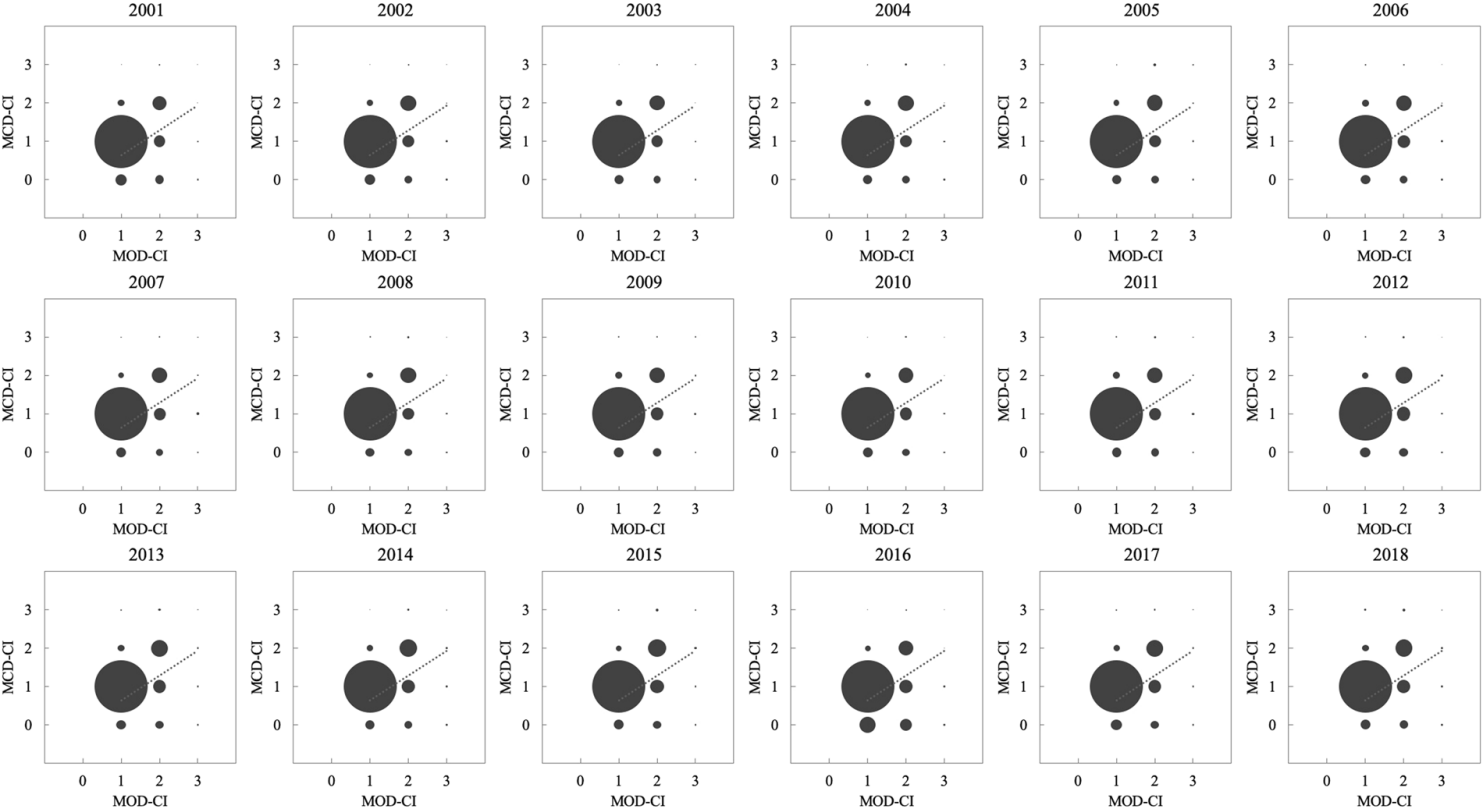
Scatter plots of cropping intensity values based on our dataset and the reference MCD12Q2 dataset.

We also examined our dataset’s accuracy by generating scatterplots of the cropping intensity values based on the pixel statistics between the GCI dataset and the reference MCN dataset for each year. The larger the bubble is, the more pixels there are (see Fig. 6). The linear fitting line and the R-squared value are used as supplementary indicators to estimate the accuracy. Overall, the GCI results are consistent with those of the MCN dataset, with an average R-squared value of 0.55 and a fitting line slope close to 1, indicating that our cropping intensity distribution maps could provide reliable estimations over the worldwide pixels based on the confirmation from MCD12Q2.

**Fig. 7:**
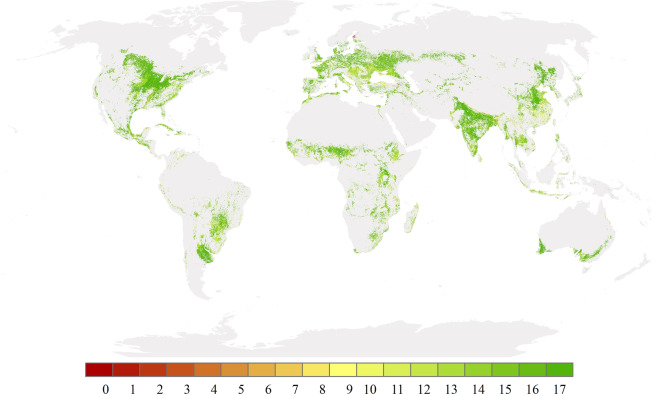
Average annual change difference between the GCI and MCN datasets from 2001 to 2018. The number indicates the year in which different changes were calculated between the GCI and MCN dataset in these 18 years.

To analyze the cropping intensity changes, we compared the difference in the annual change between the GCI and MCN datasets from 2001 to 2018. The annual difference in these changes from 2001 to 2018 is shown in Fig. S5, and the average annual change difference is also calculated (see Fig. 7). Nearly 70% of the cropping intensity area presents consistent annual changes between the GCI and MCN datasets. More than 97% of the cropland area showed a consistent change over 10 considered years. The reasons for the inconsistent changes may be that the MCN dataset focuses not only on the crops but also on the growing season, number of pastures, etc. Additionally, the accuracies of both datasets affect the results of annual change difference monitoring.

### Comparison of the results with the FAOSTAT dataset

**Fig. 8:**
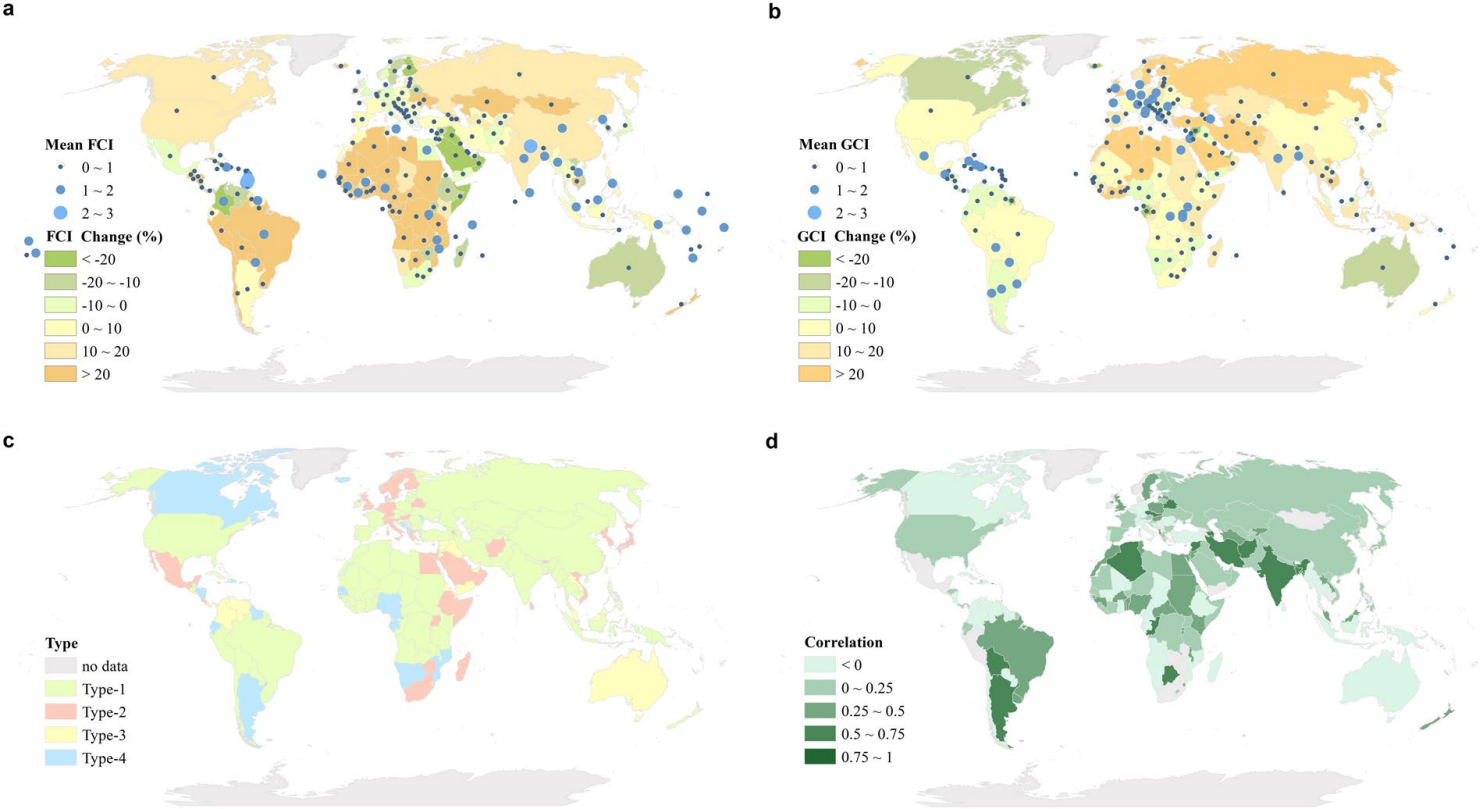
(**a**) The average FCI and FCI change ratio from 2001 to 2019 at the country level; (**b**) the average GCI and GCI change ratio from 2001 to 2019 at the country level; (**c**) countries with different FCI and GCI trend patterns; and (**d**) Pearson correlation coefficients between FCI and GCI at the country level from 2001 to 2019 with a significance level of 0.05.

Considering that all the cropland-related datasets are usually used to make decisions regarding agricultural investments and policies, determining whether this dataset is consistent with agricultural statistics represents a significant evaluation of the dataset’s application^31,32^. Using FAOSTAT, we compared the statistical cropping intensity and the average statistics of our cropping intensity dataset at the country level. The average cropping intensities from 2001 to 2019 are almost consistent between FAOSTAT (FCI hereinafter) and our results (GCI), as shown in Fig. 8(a,b). The blue points represent the intensity for every country, suggesting an overestimation by our dataset in Europe but underestimations in Asia, Africa, and Latin America. The background of every country is the FCI and GCI change ratios from 2001 to 2019, represented in Fig. 8(a,b) by the green and yellow colors, respectively. Generally, the change ratios of FCI and GCI are nearly the same, especially in some agricultural powerhouse countries, indicating that our estimations are consistent with those of FCI in terms of cropping intensity changes.

Moreover, based on the temporal evolution patterns of both FCI and GCI, we divided all countries into four types (see Fig. 8(c)): Type-1 refers to countries with increasing cropping intensities indicated by both GCI and FCI (see the green color); Type-2 denotes countries with increasing trends indicated by GCI but decreasing trends indicated by FCI (see the red color); Type-3 represents countries with decreasing trends indicated by both datasets (see the yellow color); and Type-4 comprises countries with decreasing trends indicated by GCI but increasing trends indicated by FCI (see the blue color). The change trends, shown as percentages from 2001 to 2019, are nearly the same for FCI and GCI, as shown by the mostly green and yellow colors in Fig. 8(c), although the magnitudes of these changes are different. Luckily, FCI and GCI are positively correlated almost globally (see Fig. 8(d)), demonstrating the coherence of our results in another dimensionality, although these two datasets used different approaches to generate their corresponding cropland data.

### Limitations of the global cropping intensity dataset

Our global cropping intensity dataset provides a brief and rapid way to update annual global results. It offers a high global accuracy on average but cannot represent fine-resolutions smallholder farms. In addition, we introduced sixth-order polynomial fitting to monitor the cropping intensity on a scale from 1 to 3, leading to estimates of more than three cropping cycles being omitted. Additionally, the threshold setting was based on previous research. Using only one set of uniform parameters to train the global cropping intensity model could result in detailed inaccuracies. On the other hand, the remote sensing data used in this research, the MODIS-EVI data, are not of good quality due to tropical cloudiness, and the EVI interpolation method utilized in this study cannot compensate for EVI losses across multiple consecutive days, affecting the overall results. Users need to consider these shortcomings and use this dataset based on the quality band.

## Usage Notes

Information on the global cropping intensity has received growing concern in the agriculture field, and annual dynamics have become a new trend considered in global monitoring^33^. In this study, we presented an annual dynamic global cropping intensity dataset covering the period from 2001 to 2019 at a 250-m resolution with an overall average accuracy of 89%. The global cropping intensity dataset was packaged in the GeoTIFF file type, with the quality control band in the same format. The dataset was designed so that potential users can easily visualize and analyze the dataset for their respective further research purposes using GIS software. This dataset could help researchers explore cropping potentials based on existing croplands, guide land use plans, adjust the agricultural structure, and coordinate the food trade^34,35^. This dataset fills the vacancy of global-scale, medium-resolution annual cropping intensity data, providing an improved map for further global yield estimations and food security analyses.

## Supplementary information

Supplementary Information

## Data Availability

All code used in this study is available at GitHub: https://github.com/rs-dl/CropIntensity.
