# Supplementary material for: Annual dynamic dataset of global cropping intensity from 2001 to 2019
Source: Sci Data. 2021 Oct 28;8:283. doi: 10.1038/s41597-021-01065-9 (PMC8553865; doi:10.1038/s41597-021-01065-9)
Supplement: Supplementary file 1 — Supplementary Information [file 41597_2021_1065_MOESM1_ESM.docx]

**Supplementary**

[Fig.S1. The distribution of global percentage map referring to Lu et al., 2020. 2](#_Toc78710665)

[Fig.S2. An example time series for original EVI and filtered EVI. 3](#_Toc78710666)

[Fig.S3. Spatial distribution and composition of global cropping intensity from 2001 to 2019. 5](#_Toc78710667)

[Fig.S4. Examples for (a) increasing cropping intensity and (b) decreasing cropping intensity. 6](#_Toc78710668)

[Fig.S5. Annual change difference between GCI and MCN from 2001 to 2018. 8](#_Toc78710669)


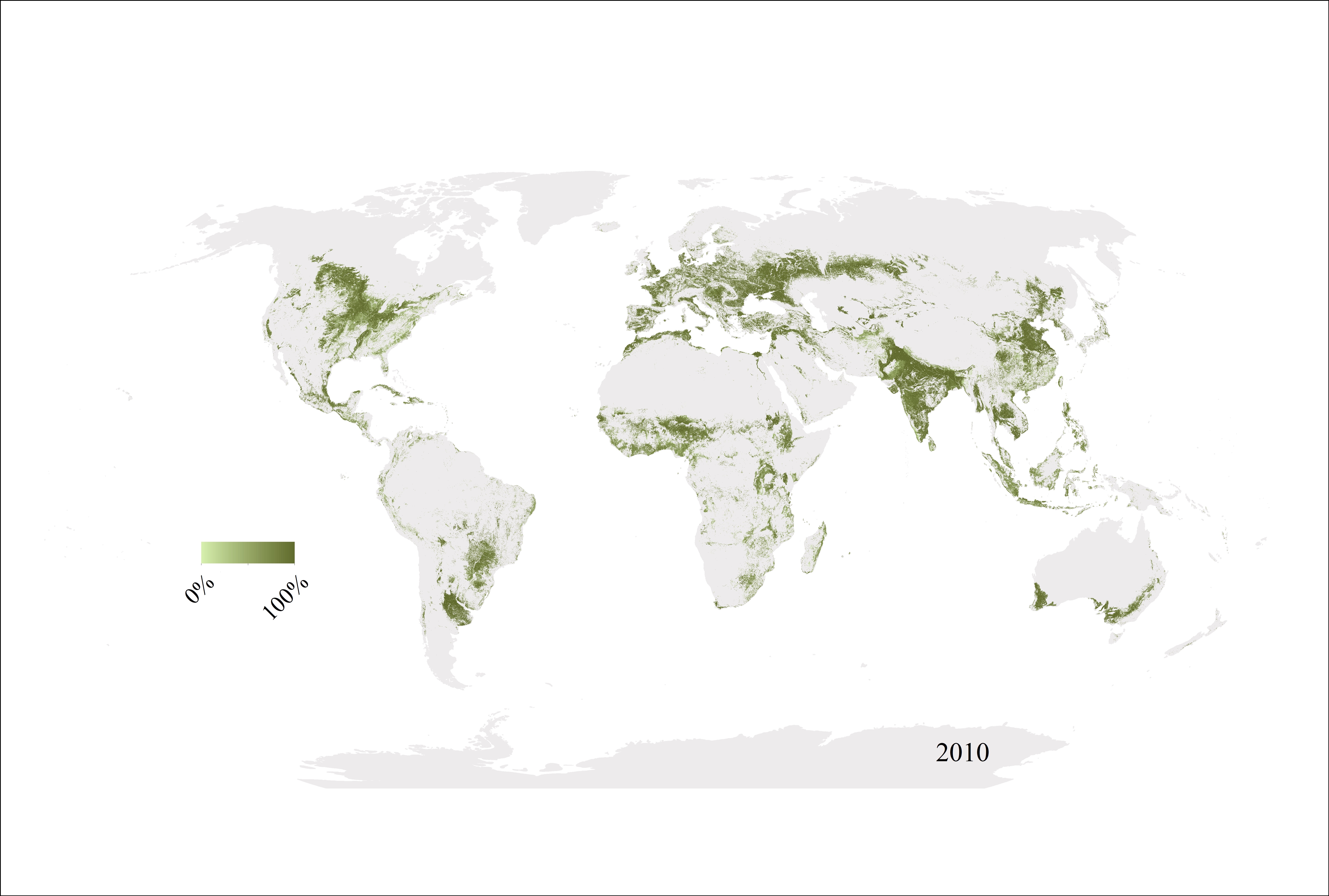


# Fig.S1. The distribution of global percentage map referring to Lu et al., 2020.

**
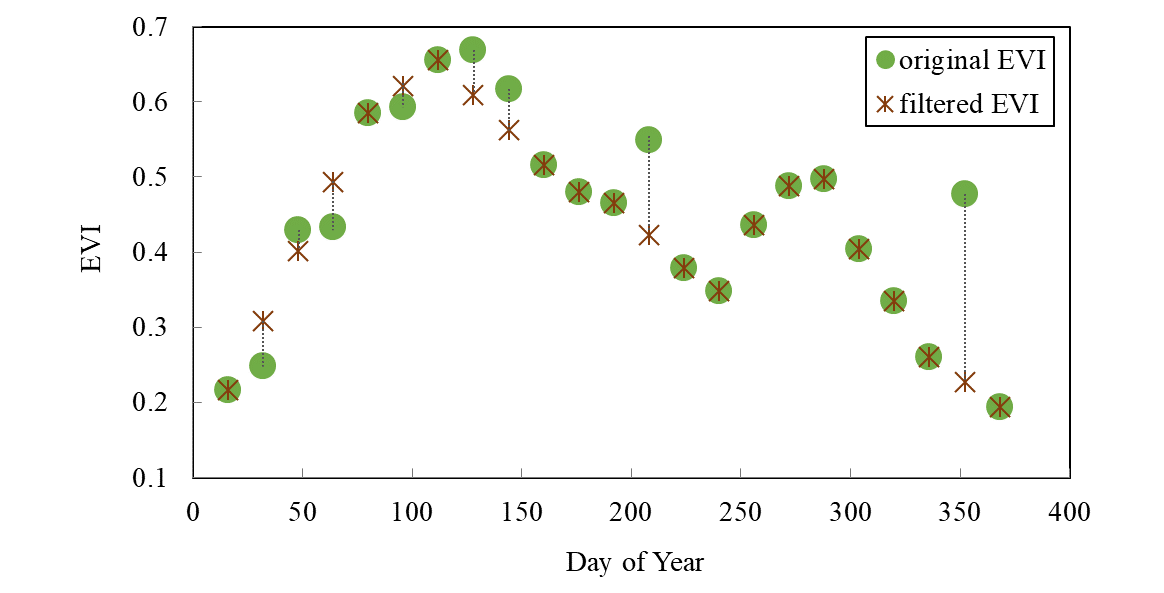
**

# Fig.S2. An example time series for original EVI and filtered EVI.

# Fig.S3. Spatial distribution and composition of global cropping intensity from 2001 to 2019.


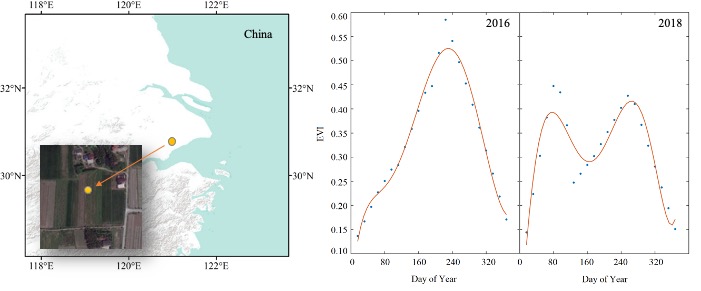


**(a)**

**
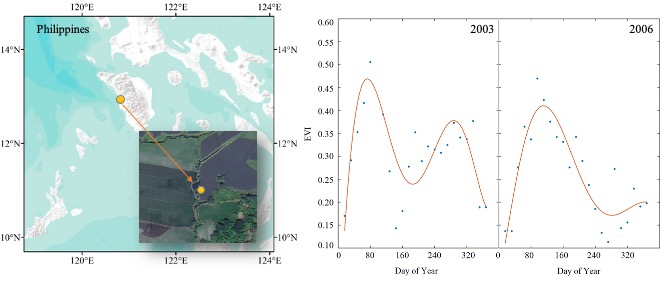
**

**(b)**

# Fig.S4. Examples for (a) increasing cropping intensity and (b) decreasing cropping intensity.


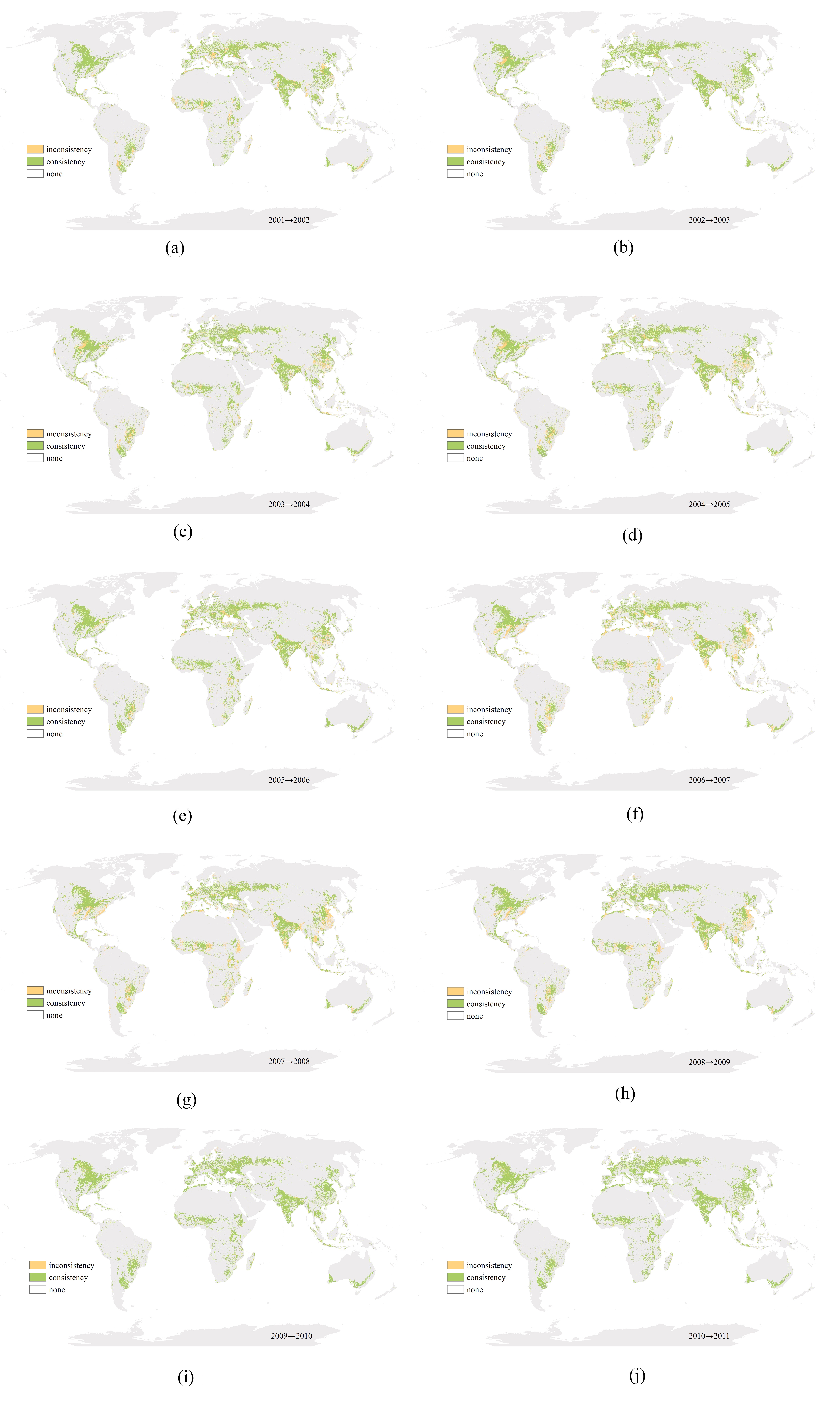


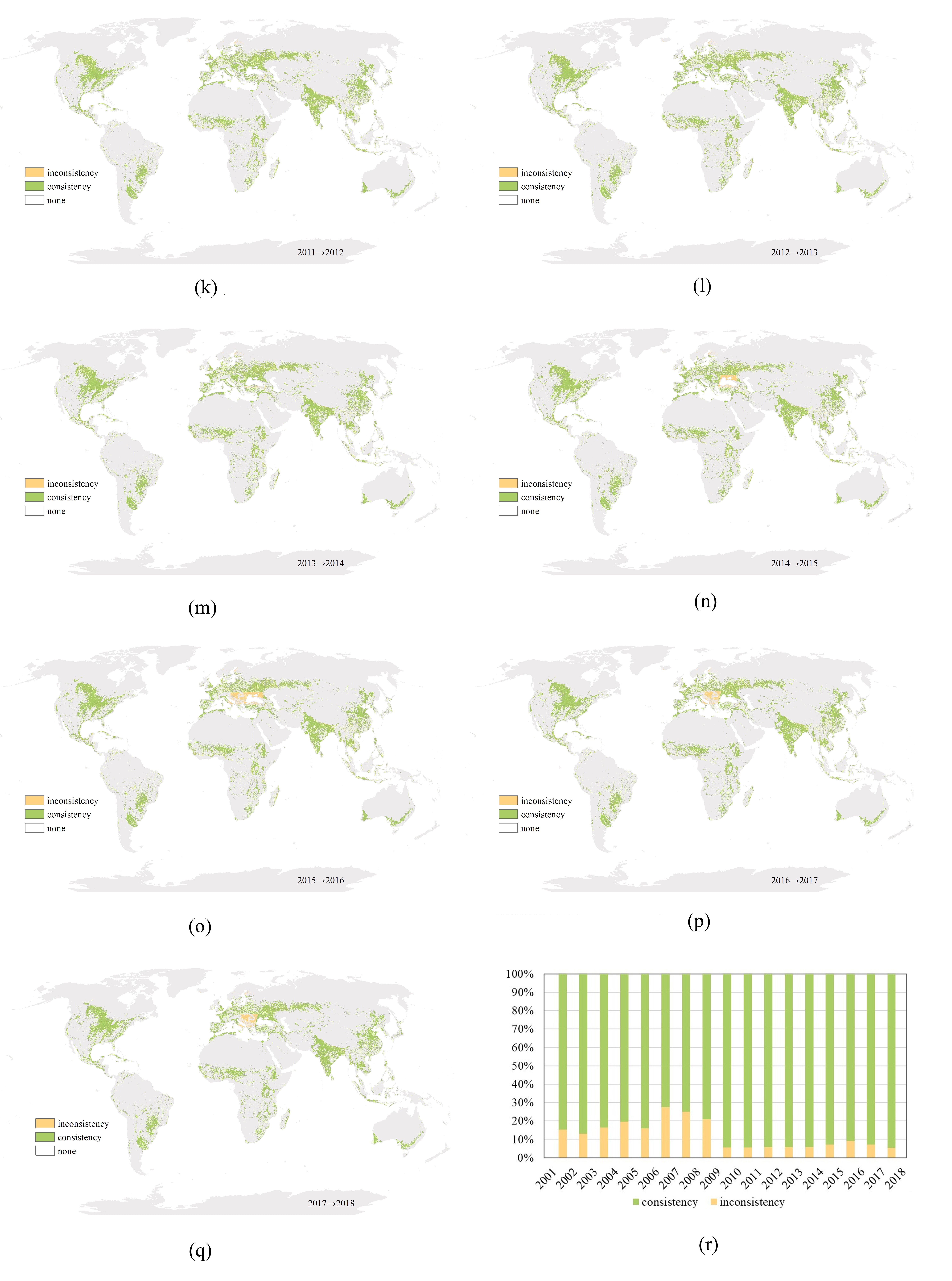


# Fig.S5. Annual change difference between GCI and MCN from 2001 to 2018.
